# Future Directions of Marine Myxobacterial Natural Product Discovery Inferred from Metagenomics

**DOI:** 10.3390/md16090303

**Published:** 2018-08-29

**Authors:** Ronald Garcia, James J. La Clair, Rolf Müller

**Affiliations:** 1 Department of Microbial Natural Products (MINS), Helmholtz Institute for Pharmaceutical Research Saarland (HIPS)—Helmholtz Centre for Infection Research (HZI), Campus E8 1, 66123 Saarbrücken, Germany; ronald.garcia@helmholtz-hips.de; 2 German Center for Infection Research (DZIF), Partner site Hannover-Braunschweig, Inhoffenstrasse 7, 38124 Braunschweig, Germany; 3 Xenobe Research Institute, P.O. Box 3052, San Diego, CA 92163-1052, USA; i@xenobe.org; 4 Department of Pharmacy, Saarland University, Campus E8 1, 66123 Saarbrücken, Germany

**Keywords:** marine myxobacteria, metagenomics, uncultured, halophilic organisms, anaerobic, 16S rRNA, *Sorangiineae*, *Nannocystineae*, *Sandaracinaceae* related clades, marine myxobacterial clusters

## Abstract

Over the last two decades, halophilic (organisms that thrive at high salt concentrations) and halotolerant (organisms that have adapted to high salt concentrations) myxobacteria emerged as an important source of structurally diverse secondary metabolites from the marine environment. This review explores the advance of metagenomics analysis and 16S rRNA gene phylogeny of the cultured and uncultured myxobacteria from marine and other salt-environments up to July 2018. The diversity of novel groups of myxobacteria in these environments appears unprecedented, especially in the *Sorangiineae* and *Nannocystineae* suborders. The *Sandaracinaceae* related clade in the *Sorangiineae* suborder seems more widely distributed compared to the exclusively marine myxobacterial cluster. Some of the previously identified clones from metagenomic studies were found to be related to the *Nannocystineae* suborder. This understanding provides the foundation for a vital, unexplored resource. Understanding the conditions required to cultivate these yet “uncultured” myxobacteria in the laboratory, while a key next step, offers a significant potential to further expand access to diverse secondary metabolites.

## 1. Introduction

The search for marine myxobacteria began over 50 years ago with the first reports appearing in the 1950s [1,2,3]. It took until 1998 that the first truly obligate halophilic and halotolerant groups were reported [4,5,6,7,8,9]. Interest in these species has arisen due to their profound ability to produce secondary metabolites that can serve as leads for drug discovery efforts [10]. Myxobacteria have since been shown to inhabit the marine and estuarine environments often found in sediments or on seagrass and algae [4,5,6,7,8]. Whether they are transient or truly originate from the sea, phylogenetic classifications allow one to determine if a given strain is a halotolerant or halophilic myxobacterium [11]. Strains belonging to the *Cystobacterineae*-type are predominantly non-halophilic except for halotolerant strains of *Myxococcus xanthus*, *Myxococcus virescens*, *Myxococcus macrosporus* [12], *Myxococcus fulvus* (strain HW-1) [13], *Angiococcus*, *Corallococcus*, and *Cystobacter* [9]. Isolated myxobacterial strains belonging to the suborder *Nannocystineae* are unique in that they grow in a wide range of salinity, and hence comprise the obligate halophilic genera *Haliangium*, *Enhygromyxa*, and *Plesiocystis*.

## 2. Phylogenetic Analysis of Cultured Marine- and Estuarine-Derived Myxobacteria 

**Figure 1:**
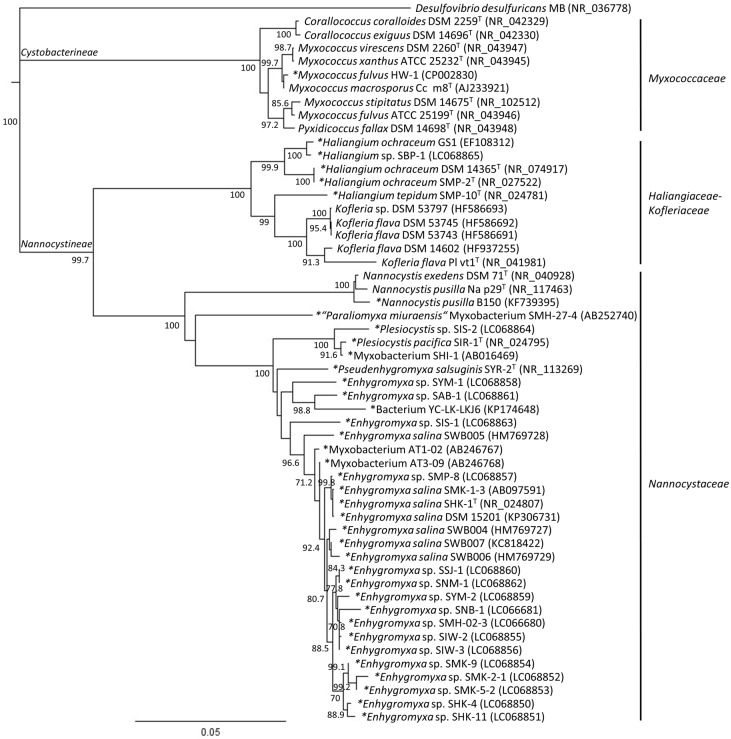
A Neighbor-joining phylogenetic tree inferred from the 16S rRNA gene sequence data depicting the position of the cultured myxobacteria in the families *Myxococcaceae*, *Haliangiaceae-Kofleriaceae*, and *Nannocystaceae* derived from saline environments (marked with asterisk). The bar represents 50 nucleotide substitutions per 1000 sites. Bootstrap values greater than 60% are shown in the nodes (based on 1000 replications). The sequence of *Desulfovibrio desulfuricans* strain MB was used as an outgroup to root the tree. ^T^ denotes type strain. Genbank accession numbers are provided in parenthesis. A complete list of the species evaluated has been provided in Table 1.

To date, only five species (*Enhygromyxa salina*, *Haliangium tepidum*, *H. ochraceum*, *Plesiocystis pacifica*, *Pseudenhygromyxa salsuginis*), belonging to four new genera of marine and estuarine-derived myxobacteria, have been validly described taxonomically [5,6,7,8]. Each of these five strains belong to the *Nannocystineae* suborder [14]. Application of the BLAST algorithm to identify similar sequences revealed that several additional strains (e.g., Myxobacterium AT1-02, Genbank AB246767; Myxobacterium AT3-09, Genbank AB246768; Bacterium YC-LK-LKJ6, Genbank KP174648) appear to be related to these taxa. That noted, phylogenetic analysis of cultured strains found by BLAST searching reveals a high diversity in the genus *Haliangium*, *Plesiocystis*, and *Enhygromyxa*, with the latter demonstrating the largest number of isolates (Figure 1, Table 1). Strains AT1-02, AT3-09, and YC-LK-LKJ6 cluster with the *Enhygromyxa* clade, while other strains such as SHI-1 emerged within the *Plesiocystis* cluster. Myxobacterium strain SMH-27-4 has been suggested as a novel genus and species, *Paraliomyxa miuraensis* [15,16]. On the other hand, the halotolerant and genome sequenced strain *Myxococcus fulvus* HW-1 [13] appears to be closely related to *Myxococcus macrosporus* based on 16S rRNA gene sequence phylogeny. So far, the culturally described halotolerant myxobacteria were only known in *Nannocystineae* and *Cystobacterineae* suborders [7,9,12]. However, obligate halophilic strains were exclusively found in *Nannocystineae* [5,6,8]. With the ongoing expanse in this field, it is very likely that new isolates will reveal novel species and genera in *Nannocystineae*.

## 3. Diversity of Myxobacteria in the Suborder *Sorangiineae* as Inferred from Metagenomic Analyses 

**Figure 2:**
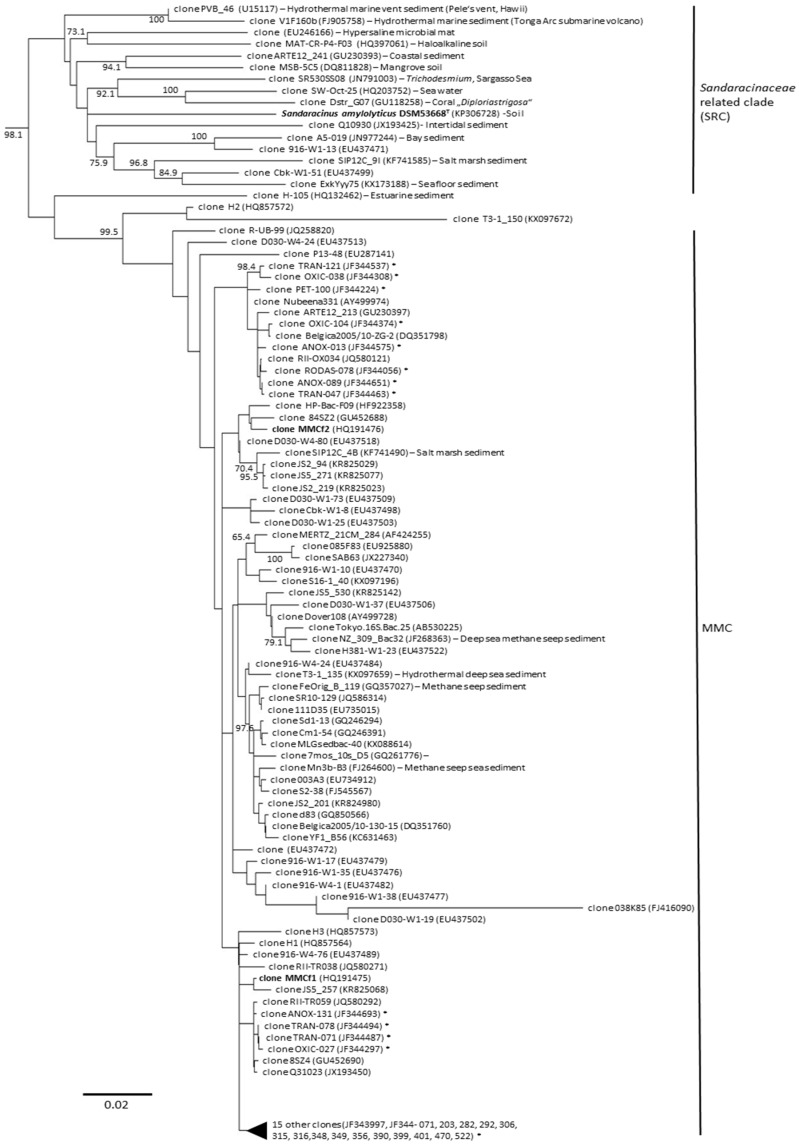
A phylogenetic tree inferred from the 16S rRNA gene sequence acquired from metagenomic analyses depicting the diversity of myxobacteria in *Sorangiineae* suborder observed in salt-containing environments. Unless indicated, the sequences were obtained from marine sediments. The GenBank accession number is indicated in parenthesis. JF34 (marked in asterisk) represents bacteria that likely grow anaerobically. The bar represents 20 nucleotide substitutions per 1000 sites. Bootstrap values greater than 60% are shown in the nodes (based on 1000 replications).

Previous reports have described the diversity of myxobacteria from the marine environment including the exclusive marine myxobacterial cluster (MMC) [17]. The MMC was detected in 6–60% salinity (sea salt content) indicating their occurrences in brackish (an environment with mixture of fresh and sea water) and marine (referring to the sea or ocean), but not in hypersaline environments. Since this report, more sequences including publicly-deposited JF34-series (GenBank JF344537), KR82-series (GenBank KR825029), and KX0-series (GenBank KX097196) appeared to belong to the MMC cluster. Phylogenetic analysis based on the 16S rRNA gene sequencing revealed that many of these sequences clustered with MMC (Figure 2). Interestingly, many of these clones in the MMC were hypothesized to have an anaerobic lifestyle including AT-s3-66 (GenBank AY225609) and AT-s3-60 (GenBank AY225609) [18]. In addition, clones of the JF34-series clone ANOX-131 (GenBank JF344693) or clone ANOX-089 (GenBank JF344651) [19] were determined to be closely related to MMCf1 and MMCf2 (Figure 2). Since many of these clones were derived from deep sea, methane seeps, and polluted hydrocarbon marine sediments, MMC may potentially comprise an anaerobic group of marine myxobacteria.

Interestingly, *Sandaracinaceae* related clade (SRC), a neighboring clade to MMC, was shown to be widely distributed in nature (Figure 2). SRC include some of the hydrothermal marine clones PVB_46 (GenBank U15117), described from Pele’s vent in Hawaii, and clones V1F160b (GenBank FJ905758) from the Tonga Arc sub-marine volcano [20]. SRC can also be found in coral, marine cyanobacterial blooms, marine seafloor sediments, sea water, mangrove sediments, hypersaline microbial mats, haloalkaline soil, and salt marsh sediment. They have also been observed in terrestrial soils. Members of the SRC are most likely taxonomically divergent as indicated by the phylogenetic distance between its species as well as their ability to thrive in a wide array of habitats. 

Together, the MMC and SRC represent interesting clades in the *Sorangiineae* suborder. To date, only one strain (*Sandaracinus amylolyticus* DSM 53668 ^T^) has been cultivated from these clades. The diversity in these environments is perhaps comparable with soil myxobacteria in terms of their potential to represent novel genera and families. While not yet achieved, it is reasonable to expect that screening efforts, similar to those developed for soil myxobacteria, will eventually provide an effective growth medium for laboratory culturing.

## 4. Diversity of Myxobacteria in the *Nannocystineae* Suborder

Our understanding of marine myxobacteria arises from studies on strains in the suborder *Nannocystineae*. However, they only occupy a small percentage of the total diversity, as the majority remains yet to be cultivated under laboratory conditions. Understanding the “uncultured” members of this suborder lies at the forefront of current discovery efforts. Truly halophilic myxobacterial isolates are typically classified as belonging to the genera *Haliangium*, *Plesiocystis*, and *Enhygromyxa*, while the halotolerant isolates belong to the genus *Pseudenhygromyxa* in *Nannocystineae* suborder. By exploration of 16S rRNA metagenomics and phylogeny, the *Nannocystineae* clade is far more environmentally diverse when compared with *Sorangiineae*. Currently, the diversity of uncultured myxobacteria in this suborder spans from saline-alkaline soil, mangrove soil, hypersaline mats, estuarine sediments to reef, marine and deep sea sediments. Overall, the issue then arises as to how one can gain access to species that have adapted a very specific environmental niche.

**Figure 3:**
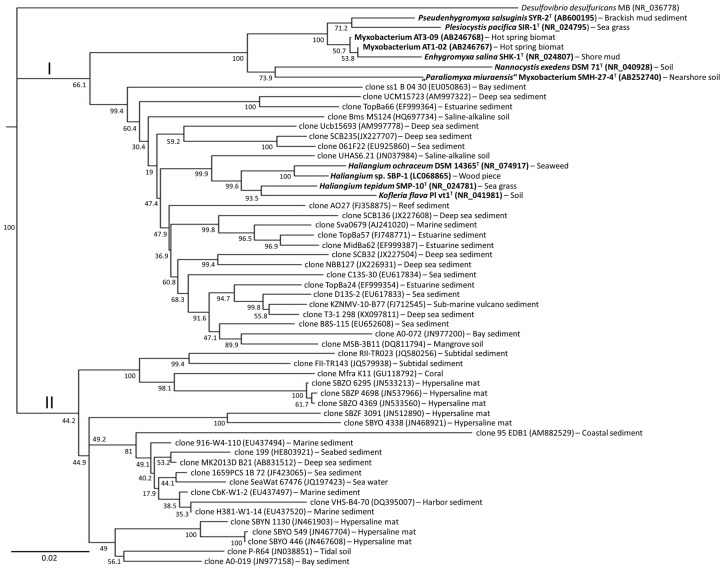
A Neighbor-joining phylogenetic tree inferred from the 16S rRNA gene sequence illustrating the diversity of myxobacteria in the *Nannocystineae* suborder from various salt-containing environments. Cultivated halotolerant or halophilic myxobacteria and their corresponding representative type strains are shown in bold-face. GenBank accession number is indicated in parenthesis. The sample source is shown after the accession number. The bar represents 20 nucleotide substitutions per 1000 sites. Bootstrap values are shown in the nodes based on 1000 replications. The sequence of *Desulfovibrio desulfuricans* strain MB was used as an outgroup to root the tree.

As shown in Figure 3, this *suborder* is divided into major clades I and II. Clade I includes members of the isolated myxobacterial strains in the genera *Enhygromyxa*, *Pseudenhygromyxa*, *Nannocystis*, *Haliangium*, and *Kofleria*. It also contains several isolates that have not yet been validated taxonomically. A large portion of the clade I cluster is comprised of yet uncultured species in the *Haliangium-Kofleria* subclade. This subclade contains the highest level of diversity and arises from deep sea [21,22] and estuarine sediments [23]. Clade II differs from clade I in the fact that not a single isolate has yet been cultured. Clade II species are found throughout a wide variety of different saline ecosystems [19,24,25,26,27,28]. 

## 5. Characteristics of Novel Marine Myxobacteria and Parameters to Consider for Their Cultivation

Based on metagenomics data and phylogenetic classification, uncultured marine myxobacteria exhibit some of the following characteristics. 

### 5.1. Structural Features: Rod-Shaped Cells with Blunted Ends 

**Figure 4:**
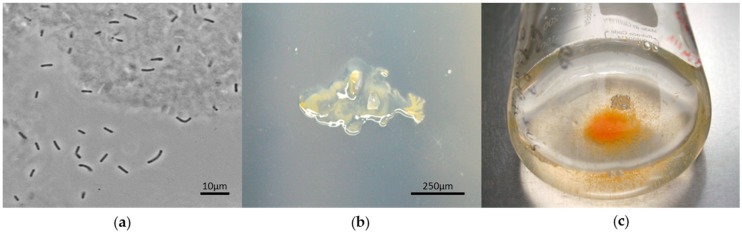
Predicted growth morphology characteristics of a marine myxobacteria in *Sorangiineae* suborder. The species name has not yet been assigned. (**a**) Phase-contrast photomicrograph of vegetative rod cells with blunted ends, (**b**) Stereophotograph of a colony showing the pattern of agar degradation, (**c**) Growth in liquid medium containing sea salts showing some cell aggregates after shaking.

Strains classified within *Sorangiineae* and *Nannocystineae* typically exhibit rod-shaped cells with blunted ends (Figure 4a). These suborders are unique for this type of cell shape. Such cells can be differentiated from strains belonging to the *Cystobacterineae* suborder [29], for which vegetative cells appear as thinner flexuous rods. The uncultured marine *Sorangiineae* and *Nannocystineae* cells may have adapted to their aquatic environment to become motile. 

### 5.2. Reproductive Features: Reduction in Forming Fruiting Bodies 

Studies to date have shown that fruiting body formation in select halotolerant strains is supported by up to 60% *w/v* seawater (equivalent to 2% *w/v* salt in solid medium) [9]. Other studies, however, have shown that fruiting body-bearing spores from halophilic strains including *Haliangium tepidum* [8,30], *Enhygromyxa salina* [5,31], and *Plesiocystis pacifica* [6,31] can still be observed at sea water salinity (~3.5% *w/v*). Isolation of novel myxobacteria from salt environments should not rely completely on the appearance of fruiting bodies, as this structure may not develop. Further studies have shown that strains belonging to the SRC may not form fruiting bodies as exemplified by *Sandaracinus* within this clade [32].

### 5.3. Chemical Predation and Defense: Lack of Microbial Predation and Cellulose Degradation but Agar Degradation

Based on metagenomic analysis of salt-containing environments, uncultured myxobacteria appear to belong to the suborders *Sorangiineae* (Figure 2) and *Nannocystineae* (Figure 3). Since these suborders are known for agar degradation, one might expect that these myxobacteria may also share this characteristic (Figure 4b). Microbial lysis is known in myxobacteria, however, this trait may be lost in samples derived from deep sea ecosystems. Since strains have yet to be obtained from deep sea sediments, it is not known whether these myxobacteria can prey on other microorganisms. So far, none of the *Nannocystineae* isolated to date degrade cellulose, an observation, which is commonly found in terrestrial strains. 

### 5.4. Requirement for Salinity: A Need for Salt and Associate Metal Cations 

Marine-derived myxobacteria differ from terrestrial isolates primarily in their salt tolerance (Figure 4c). They seem to have adapted a system to adjust to saline environments using osmolytes such as ectoine, hydroxyectoine, and glycine betaine [33]. In halotolerant *Myxococcus* strains, salt tolerance was speculated to be acquired by horizontal *hdsp* gene transfer [34], while *Plesiocystis pacifica* SIR-I strain makes use of amino acids as osmoprotectant [35]. In addition to modulation of salt levels, isolated marine myxobacteria typically require the addition of cations such as Ca^2+^, Mg^2+^, and K^+^ to support growth. 

### 5.5. Temperature Regulation: Growth Possible in Wider Temperature Range

To date, the isolated marine and estuarine-derived myxobacteria grew at a wider temperature range (5–45 °C) [5,8,31] than terrestrial species. While the aqueous environment of marine species plays a key role in temperature regulation, it is likely that they have highly adapted and evolved to match the temperature of their local environment. For instance, tropical sandy beaches will likely contain marine myxobacteria, which can resist higher temperatures [8] than those collected in frigid seas in the North Atlantic [17].

### 5.6. Life Cycle: Potentially Slow Growth

Since metagenomic and phylogenetic data revealed that novel myxobacteria in salt-containing environments were positioned in the *Sorangiineae* and *Nannocystineae* suborders, it might be expected that they grow much slower when compared with most terrestrial counterparts. In general, cultured members of these suborders may be recognized by their growth after weeks of incubation in the isolation set-ups. One might speculate that these organisms may be microaerophilic, anaerobic, and utilize different carbon sources such as methane, since many of these clones were derived from hydrothermal vents, sub-marine volcanic ash, and deep sea seep sediments.

## 6. Can Marine Myxobacteria Access Novel Bioactive Secondary Metabolites? 

Marine-derived myxobacteria have already demonstrated a strong potential to produce natural products with distinct scaffolds [36,37,38,39,40,41]. Many of these materials display unique structure features as exemplified by the enhygrolides [36], enhygromic acid [37], haliamide [38], haliangicin [39,40], miuraenamide [15,16], salimabromide [41], and the salimyxins [36] (the structures of these materials are provided in the accompaning manuscript [10]). Many of these materials have demonstrated potent biological activity [42]. For instance, enhygrolide A was shown by the König laboratory to inhibit the growth of the Gram-positive bacterium at microgram concentration (MIC, 4 µg mL^−1^ against *Arthrobacter cristallopoietes*) [36]. Other studies including those on enhygromic acid (IC_50_ value of 46 µM, B16 melanoma cells) [37] or haliamide (12 µM, HeLa-S3 cells) [38] have shown activities against the proliferation of tumor cells. Studies on haliangicin have identified antifungal activities with minimal inhibitory concentration (MIC) values comparable to amphotericin and nystatin [40]. While the modes of action of these compounds still remain unexplored, morphology screening efforts identified actin filament stabilizing activity associated with miuraenamide A [43]. While early on, the evidence collected to date indicates that unique bioactive scaffolds can be obtained from marine- and estuarine-derived myxobacteria.

A large percentage of marine myxobacterial strains remain uncultured to-date, as access to these strains remains challenging under laboratory conditions. Furthermore, the potential to produce novel compounds by marine myxobacteria is also reflected in the diversity of the biosynthetic gene clusters encoded in the few available genome sequences [44,45]. Previous studies have shown novel Type I polyketide synthase genes that can be found in marine myxobacteria [46], which indicates the potential of these organisms to produced novel compounds. A further description of the unique complexity of these synthases and their associated natural products has recently been reviewed [10].

## 7. Conclusions

Myxobacteria appear to be ubiquitous and diverse in nature, and are not just terrestrial but found in marine, estuarine, and a variety of other saline ecosystems. Metagenomic analyses indicate that the marine myxobacteria identified to date occupy only a small portion of the potential *Nannocystineae* phylogenetic tree. This suggests that a vast diversity of species are unexplored. The development of new methods and tools to culture these species would unveil a yet untapped and robust potential. This, combined with the well-established ability of myxobacteria to produce unique and bioactive molecules, identifies the suborder *Nannocystineae* as an important resource for future therapeutic evaluation. The suborder *Sorangiineae* also holds great promise for natural product discoveries. Currently, not a single species of the exclusively MMC have been isolated and cultured. In addition, the SRC appears interesting since it is not well explored and also seems distributed through a vast array of marine environments. Based on metagenomic and phylogenetic analysis, those clones derived from marine deep-sea sediments may return extremophilic myxobacteria with access to halophilic, anaerobic, microaerophilic, piezophilic, or psychrophilic properties. Finding the requirements for salinity, salts, temperature, and dissolved gasses is obviously necessary. Simulating the conditions of an organism’s natural environment is likely key to the successful cultivation of marine myxobacteria. This observation suggests that we explore the development of laboratory culturing systems that operate at sea. Here, one can envision the development of robotic devices that mimic the natural conditions in marine ecosystems such as that in a sponge [47]. Recent discoveries clearly indicate that marine myxobacteria are under explored group of organisms that offer tremendous potential for secondary metabolite and molecular mining, either through the advance of modern devices or classical approaches.

## Figures and Tables

**Table 1:** List of strains and their GenBank accession number.

Bacterial Strains	GenBank Accession Number
Bacterium YC-LK-LKJ6	KP174648
*Corallococcus coralloides* DSM 2259 ^T^	NR_042329
*Corallococcus exiguus* DSM 14696 ^T^	NR_042330
*Desulfovibrio desulfuricans* MB	NR_036778
*Enhygromyxa* sp. SAB-1	LC068861
*Enhygromyxa salina* SHK-1 ^T^	NR_024807
*Enhygromyxa* sp. SHK-4	LC068850
*Enhygromyxa* sp. SHK-11	LC068851
*Enhygromyxa* sp. SIS-1	LC068863
*Enhygromyxa* sp. SIW-2	LC068855
*Enhygromyxa* sp. SIW-3	LC068856
*Enhygromyxa* sp. SMH-02-3	LC066680
*Enhygromyxa salina* SMK-1-3	AB097591
*Enhygromyxa* sp. SMK-2-1	LC068852
*Enhygromyxa* sp. SMK-5-2	LC068853
*Enhygromyxa* sp. SMK-9	LC068854
*Enhygromyxa* sp. SMP-8	LC068857
*Enhygromyxa* sp. SNB-1	LC066681
*Enhygromyxa* sp. SNM-1	LC068862
*Enhygromyxa* sp. SSJ-1	LC068860
*Enhygromyxa* sp. SYM-1	LC068858
*Enhygromyxa* sp. SYM-2	LC068859
*Enhygromyxa salina* DSM 15201	KP306731
*Enhygromyxa salina* SWB004	HM769727
*Enhygromyxa salina* SWB005	HM769728
*Enhygromyxa salina* SWB006	HM769729
*Enhygromyxa salina* SWB007	KC818422
*Haliangium* sp. SBP-1	LC068865
*Haliangium ochraceum* GS1	EF108312
*Haliangium ochraceum* DSM 14365 ^T^	NR_074917
*Haliangium ochraceum* SMP-2 ^T^	NR_027522
*Haliangium tepidum* SMP-10 ^T^	NR_024781
*Kofleria* sp. DSM 53797	HF586693
*Kofleria flava* DSM 53745	HF586692
*Kofleria flava* DSM 53743	HF586691
*Kofleria flava* DSM 14602	HF937255
*Kofleria flava* Pl vt1 ^T^	NR_041981
Myxobacterium SHI-1	AB016469
Myxobacterium SMH-27-4 (“*Paraliomyxa miuraensis*”) *	AB252740
Myxobacterium AT1-02	AB246767
Myxobacterium AT3-09	AB246768
*Myxococcus fulvus* HW-1	CP002830
*Myxococcus fulvus* ATCC 25199 ^T^	NR_043946
*Myxococcus macrosporus* Cc m8 ^T^	AJ233921
*Myxococcus stipitatus* DSM 14675 ^T^	NR_102512
*Myxococcus virescens* DSM 2260 ^T^	NR_043947
*Myxococcus xanthus* ATCC 25232 ^T^	NR_043945
*Nannocystis exedens* DSM 71 ^T^	NR 040928
*Nannocystis pusilla* Na p29 ^T^	NR_117463
*Nannocystis pusilla* B150	KF739395
*Plesiocystis pacifica* SIR-1 ^T^	NR_024795
*Plesiocystis* sp. SIS-2	LC068864
*Pseudenhygromyxa salsuginis* SYR-2 ^T^	NR_113269
*Pyxidicoccus fallax* DSM 14698 ^T^	NR_043948

* Tentative name proposal based on reference 15 and 16 and ^T^ denotes type strain.
